# Identifying the natural reserve area of *Cistanche salsa* under the effects of multiple host plants and climate change conditions using a maximum entropy model in Xinjiang, China

**DOI:** 10.3389/fpls.2022.934959

**Published:** 2022-08-17

**Authors:** Minghao Shao, Jinglong Fan, Jinbiao Ma, Lei Wang

**Affiliations:** ^1^National Engineering Technology Research Center for Desert-Oasis Ecological Construction, Xinjiang Institute of Ecology and Geography, Chinese Academy of Sciences, Urumqi, China; ^2^University of Chinese Academy of Sciences, Beijing, China; ^3^Taklimakan Desert Research Station, Xinjiang Institute of Ecology and Geography, Chinese Academy of Sciences, Korla, China; ^4^State Key Laboratory of Desert and Oasis Ecology, Xinjiang Institute of Ecology and Geography, Chinese Academy of Sciences, Urumqi, China

**Keywords:** *Cistanche salsa* (C. A. Mey.) G. Beck, nature reserve, multiple host factors, climate changes, species distribution model

## Abstract

*Cistanche salsa* (C. A. Mey.) G. Beck, a holoparasitic desert medicine plant with multiple hosts, is regarded as a potential future desert economic plant. However, as a result of excessive exploitation and poaching, its wild resources have become scarce. Thus, before developing its desert economic value, this plant has to be protected, and the identification of its natural reserve is currently the top priority. However, in previous nature reserve prediction studies, the influence of host plants has been overlooked, particularly in holoparasitic plants with multiple hosts. In this study, we sought to identify the conservation areas of wild *C. salsa* by considering multiple host–plant interactions and climate change conditions using the MaxEnt model. Additionally, a Principal Component Analysis (PCA) was used to reduce the autocorrelation between environmental variables. The effects of the natural distribution of the host plants in terms of natural distribution from the perspective of niche similarities and extrapolation detection were considered by filtering the most influential hosts: *Krascheninnikovia ceratoides* (Linnaeus), Gueldenstaedt, and *Nitraria sibirica* Pall. Additionally, the change trends in these hosts based on climate change conditions combined with the change trends in *C. salsa* were used to identify a core protection area of 126483.5 km^2^. In this article, we corrected and tried to avoid some of the common mistakes found in species distribution models based on the findings of previous research and fully considered the effects of host plants for multiple-host holoparasitic plants to provide a new perspective on the prediction of holoparasitic plants and to provide scientific zoning for biodiversity conservation in desert ecosystems. This research will hopefully serve as a significant reference for decision-makers.

## Introduction

Desert economy refers to the development of industries based on local characteristics according to the unique climatic, geographical, and resource conditions of arid areas, thus driving local economic development (Fan et al., 2020; Shao et al., 2022). *Cistanche salsa* (C. A. Mey.) G. Beck, a holoparasitic plant with multiple host plants living in expansive arid areas, has the potential to become one of the potential targets for desert economic development (Editorial Committee of Flora Reipublicae Popularis Sinicae, 1990; Fan et al., 2020; Station et al., 2020). Scholars have found that all tissues of *C. salsa* comprise medicinal components that can inhibit the enzymes that are implicated in human ailments *in vitro*, including tyrosinase for skin hyperpigmentation disorders, α-glucosidase and α-amylase for diabetes, and acetyl-(AChE) and butyrylcholinesterase (BuChE) for Alzheimer’s disease (Jiang and Tu, 2009; You et al., 2016; Trampetti et al., 2019; Song et al., 2021). Additionally, because of its medicinal and economic values, natural sources of this plant have been decreasing in recent years (Sun X. et al., 2020). Hence, before developing the economic industry, we first need to protect the wild germplasm resources by identifying its natural reserves. Subsequently, industrial coordination and functional area development can be carried out according to the distribution of the natural reserves.

For natural reserve identification and distribution prediction, species distribution models (SDMs) are widely used, with the Maximum Entropy (MaxEnt) algorithm being popular due to its user-friendliness (Di Marco et al., 2018; Galante et al., 2018; Sun J. et al., 2020). Using limited records and environmental variables, the target species can be easily predicted in study areas (Moreno-Amat et al., 2015; Gherghel et al., 2018; Wiese et al., 2019; Mandakh et al., 2020). However, common mistakes, such as the spatial resolution of records and variables and the correlations between variables, which can reduce the credibility of the MaxEnt model, are also ignored by researchers (Sillero and Barbosa, 2021). Additionally, for distribution prediction, climate change is also a key factor, especially under the conditions of species extrapolation with time range shifting (Elith et al., 2010; Nguyen et al., 2022). The risk of climate change will also affect SDM accuracy and reliability, particularly in terms of the climate uncertainty of the Global Climate Model (GCM) from a single institute, so at least two or more GCMs from other institutes can be used to check or reduce the uncertainty (Araújo et al., 2005, 2019).

In addition to considering how these issues are related to the model settings, for holoparasitic plants, in particular, the effects of the host plants playing a fundamental role in its growth must be considered (Wang et al., 2009; Smith et al., 2013; da Cunha et al., 2018). Some researchers have been studying the role of host plants. In 2009, David proposed the host quality hypothesis, which is based on a summary of previous research and states that host quality is the critical factor governing the survival of parasitic plants (Watson, 2009). Follow-up studies on the host quality hypothesis have also focused on hemiparasitic plants, such as Mistletoe and Loranthaceae (Dibong et al., 2012; Sayad et al., 2017). Moreover, subsequent studies on holoparasitic and hemiparasitic plants have also shown that the host plant’s distribution, growth, and developmental characteristics have an essential influence on the distribution of parasitic plants (Hu et al., 2020; Shao et al., 2022).

However, some studies still treat holoparasitic plants, such as *C. salsa* and *C. deserticola* as non-parasitic plants, during distribution prediction and only investigate the role of the climate, soil, and other environmental factors (Li Z. et al., 2019; Liu et al., 2019; Sun X. et al., 2020). To better understand and predict *C. salsa* distribution in natural conditions and to identify the effects of host plants in natural reserves, our study aims to (1) analyze the changes in the host plant caused by climate change; (2) predict *C. salsa* distribution according to the effects of the host plant; and (3) identify the natural reserve by changing the habitat of *C. salsa* and its hosts in different scenarios. Additionally, these results will also help other researchers and local government bodies to better understand how to protect multi-host parasitic plants.

## Materials and methods

### Study area, host selection, and occurrence records

The Xinjiang Uyghur Autonomous Region comprises about one-sixth of the Chinese national territory area (totally 1,664,900 km^2^) and is the location of two large deserts (the Taklimakan desert and the Gurbantunggut desert). Its unique climate and soil conditions mean that it may experience potential economic and research development (Zhou et al., 2016; Yan et al., 2019; Yang et al., 2021). Most plants cannot live here because of the region’s extremely bad ecological environment, but it is also the habitat of some unique species that can live in high-evaporation and low-precipitation conditions (Li et al., 2011; Li and Sun, 2017). *C. salsa* is one such arid plant, and Xinjiang is its main place of origin in China (Editorial Committee of Flora Reipublicae Popularis Sinicae, 1990). Hence, understanding the plant’s natural reserves in Xinjiang is conducive to planning a long-term development schedule and research projects.

Host plants of *C. salsa* include *Kalidium foliatum* (Pall.) Moq., *K. gracile* Fenzel, *K. cuspidatum* (Ung.-Sternb.) Grub., *Reaumuria soongarica* (Pall.) Maxim., *Nitraria sibirica* Pall., and *Achnatherum splendens* (Trin.) Nevski (Editorial Committee of Flora Reipublicae Popularis Sinicae, 1990). However, new host plants such as *Suaeda physophora* Pall. and *Krascheninnikovia ceratoides* (Linnaeus) *Gueldenstaedt* have been observed in fieldwork. As such, considering the history and current conditions, these host plants should all be regarded as host factors for future *C. salsa* prediction. In the present study, occurrence records (for host plants and parasitic plants) were obtained from online databases, including the Global Biodiversity Information Facility (GBIF), Chinese Virtual Herbarium National Plant Specimen Resource Center (CVH NPSRC), National Specimen Information Infrastructure (NSII), and fieldwork records. Additionally, after the occurrence data were obtained, the data were cleaned by removing the records without coordinates and duplicate data. These data were saved as the “.csv” files with the terms “longitude” and “latitude” in the header. However, these “.csv” files cannot be used in MaxEnt modeling directly, and there is a final step to be completed after preparing the environmental variables: that is, the resolution of occurrence records should be the same as that of variables. There were 465 occurrence records in total.

### Environmental variables

The current and future climate variables are all from the WorldClim 2.1 database (including 19 bioclimatic variables and elevation) and have a spatial resolution of 2.5 Arc-min. The spatial resolution of 30 Arc-s observed in the bioclimatic variables can be downloaded for both current and future scenarios but cannot be used in this study because the resolution of the variables is extremely accurate and the resolution of the occurrence records is not as accurate (Sillero and Barbosa, 2021). Additionally, considering the area of Xinjiang, the spatial resolution of 2.5 Arc-min is enough for MaxEnt modeling. Additionally, the use of future climate simulations conducted in different institutes avoids some of the climate uncertainty resulting from a single climate model (Araújo et al., 2019), so the mean of the MIROC6, BCC-CSM2-MR, CNRM-CM6-1, and CanESM5 future climate models were used in this study and was determined using the R packages “raster” and “rasterVis” (Hijmans, 2021; Perpiñán and Hijmans, 2021). Furthermore, the soil and topographical variables shaping the distribution of arid plant species must be considered (Mod et al., 2016; Buri et al., 2017; Figueiredo et al., 2018). As such, the data of the related variables downloaded from NASA and OpenLandMap include the soil water content (at 100 and 200 cm depth), soil sand content (at 100 and 200 cm depth), soil organic carbon content (at 100 and 200 cm depth), soil texture class (at 100and 200 cm depth), and aspect and slope. All of these data have a spatial resolution that is more accurate and can be processed once reaching the spatial resolution of 2.5 Arc-min using the Google Earth Engine.

**FIGURE 1:**
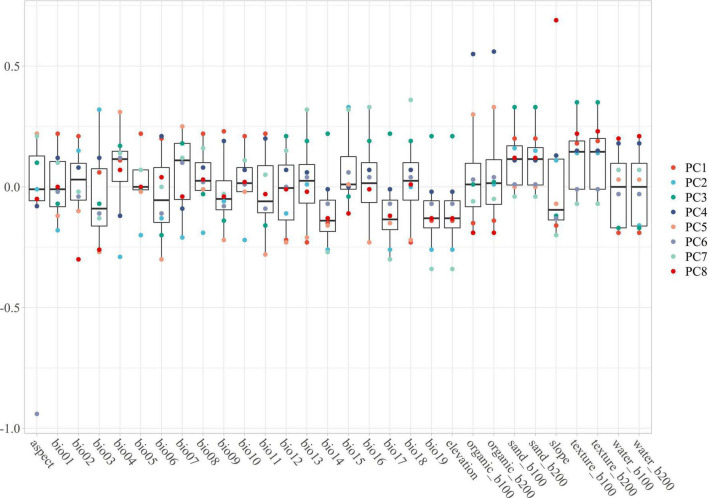
The PC1 to PC8 are containing with the main information of environmental variables. The main feature of each variable can be contained in one or more PCA, especially the outlier should pay more attention. The PC1 contains the features of bio01, bio05, bio06, bio09, bio11, bio13, and bio16; The PC2 contains the features of bio01, bio03, bio04, bio05, bio07, bio08, bio10, bio14, and bio15; The PC3 contains the features of bio12, bio14, bio17, bio19, elevation, sand_b100, sand_b200, texture_b100, and texture_b200; The PC4 contains the features of bio06, bio09, bio11, organic_b100, and organic_b200; The PC5 contains the features of bio03, bio04, bio06, bio09, bio11, bio12, bio13, bio16, bio18, organic_b100, and organic_b200; The PC6 contains the feature of aspect; The PC7 contains the features of bio13, bio14, bio15, bio16, bio17, bio18, bio19, and elevation; The PC8 contains the features of bio02, bio05, bio15, organic_b100, organic_b200, slope, water_b100, and water_b200.

Environmental variables (30 files) with the same resolution cannot be used in MaxEnt modeling directly. The use of highly correlated variables in building models without critically analyzing them will result in undesired effects (Franklin, 2010; Field et al., 2012; De Marco and Nóbrega, 2018; Sillero and Barbosa, 2021): the null hypothesis can be wrongly rejected; coefficients can change significantly and may even reverse their sign; insignificant variables can be selected; the model can suffer from over-fitting, being excessively adjusted to the data (possibly reflecting noise); and it may be not possible to correctly disentangle the response curves for each variable, as each variable will interact with others, making it difficult to obtain the actual response curves. To avoid these undesired effects observed in past research, the Pearson and Spearman correlation matrices have been used, obtaining correlation thresholds of around 0.7 or higher (Dormann et al., 2013). However, (1) collinearity effects models trained on data from one region or time and then projected to another with a different or unknown collinearity structure (Dormann et al., 2013); and (2) precipitation will always be related to temperature or elevation or both, and other variables are associated with everything else, so there are no completely uncorrelated variables (Tobler, 1970). Hence, considering that everything is related to everything and that the effect of collinearity effects change as time and climatic variables change, Principal Component Analysis (PCA) should be considered as an approach because it can transform all environmental variables into different orthogonal components while maintaining their ecological meaning (Hirzel et al., 2002). For this goal, the R package “ENMTML” was used for variable selection and variable reconstitution in the PCA approach (de Andrade et al., 2020). The raw 30 environmental variables were transformed into 8 restructured variables PC1 to PC8, which contain over 95% of the variable information (Figure 1) that could be used in model building.

### Maximum entropy modeling

There are two fundamental steps in model building: (1) the preparation and processing of occurrence records and environmental variables; and (2) the optional strategy for the research object.

For step one, the occurrence records are processed using data clean, and the variables also are processed by PCA. Before putting them together in the Maxent model “machine,” the spatial resolutions should be the same (Sillero and Goncalves-Seco, 2014). To achieve this, the R package “ENMeval” provides a tool to put records into the variable spatial raster and filter the surplus records to make one record in one spatial raster (Muscarella et al., 2014; Kass et al., 2021).

Additionally, for step two, *C. salsa* is a holoparasite plant with multiple hosts, and it is not easy to input biotic factors when predicting the performance effects of the host, and this is often ignored by other researchers (Sun X. et al., 2020). Recently, researchers have proposed an approach in which the biotic factors can be regarded as the normal environmental factors during model building to produce a predictive distribution (Shao et al., 2022). According to this approach, the model building takes place in two steps: (1) the host plant distributions are produced: in each scenario, the MaxEnt model is built with each host occurrence record and all of the processed variables (PC1 to PC8 can indicate over 95% of the influence of the original variables); and (2) the predictive distribution of *C. salsa* is produced: in each scenario, the MaxEnt model is trained using *C. salsa* records and host plant distributions (as the effect factors of parasite plant). As such, all of the processes within a specific climate condition can be included in three steps: (1) the records of the host plants and *C. salsa* and the environmental variables (PC1 to PC8) are processed; (2) the predicted distribution of the host plants is determined by factors PC1 to PC8 (these distributions are regarded as in-process factors for the next step); and (3) the predicted distribution of *C. salsa* is determined by the in-process factors (the distributions of its hosts).

This approach is clear, but there is still a question of how to choose the optional model during training. Traditionally, the AUC (area under the curve) is a good choice for optional model selection (Radosavljevic and Anderson, 2014; Kass et al., 2020). However, some researchers have pointed out that the AUC is not an optional or appropriate option for the performance measure of presence-background or presence-only ecological niche models (Lobo et al., 2008; Leroy et al., 2018; Sofaer et al., 2019; Velasco and González-Salazar, 2019). As such, for presence-only models, the Continuous Boyce index (CBI) may be more suitable (Boyce et al., 2002; Hirzel et al., 2006; Viña et al., 2010). The CBI value is between −1 and +1. The positive value means that the predictive distribution is consistent with the presented distribution, and the negative value indicates that the habitat has poor predicted quality (Hirzel et al., 2006; Sun et al., 2021). Additionally, before the final step, cross-validation must be selected. Spatial cross-validation can reduce the effects of spatial autocorrelation in the occurrence data, which avoids overly optimistic model performance due to the spatial dependence between localities that can occur through random cross-validation (Roberts et al., 2017). The R package “ENMeval” also provides some options for cross-validation, such as block, checkerboard, hierarchical checkerboard, and so on (Muscarella et al., 2014; Kass et al., 2021). Considering the area and occurrence in the study region, the hierarchical checkerboard was chosen for this study (Radosavljevic and Anderson, 2014; Roberts et al., 2017). In summary, the whole model generation process includes three main steps: (1) inputting the data, including clean species occurrence records and effect factors (for the host plants, the effect factors are the variables from PC1 to PC8, and for C. salsa, the effect factors are the distributions of its hosts); (2) producing the all possible model-parameter settings with evaluations by considering cross-validation and appropriate features [L, Q, H, LQ, LH, QH, LQH (Phillips et al., 2017)]; and (3) selecting the optional model according to the maximum CBI.

Additionally, after models are produced, the extrapolation risk in prediction training should be estimated due to the distribution models being produced from the limited data in the limited zone to the extent zone (King and Zeng, 2007; Mesgaran et al., 2014; Mannocci et al., 2017, 2018). In this step, the “dsmExtra” package in R is used to produce the extrapolation detection (ExDet) metric (which assesses the extrapolation in environmental space and model transferability) and the percentage of data nearby (%N) (quantitatively assessing the extrapolation reliability in multivariate environmental space) (Bouchet et al., 2020). Additionally, to interpret the extrapolation assessments simply, a lower ExDet is better; a higher%N is better; a high ExDet with a low%N is the least reliable; and a low ExDet with a high%N is the most reliable (Miller et al., 2013; Bouchet et al., 2019). Additionally, to assess the effects of the host plants, the ExDet metric, and the “*I*” similarity statistic can all quantitatively evaluate this goal (Warren et al., 2008). These approaches are independent of the model training processing (Broennimann et al., 2012; Bouchet et al., 2019).

### Assessing the conservation areas

We have primarily used two principles for the delineation and identification of conservation areas: (1) the classification of the fitness class of the distribution of wild resources of *C. salsa* under current environmental conditions; and (2) the change in the habitat class of areas under future climate change conditions (Shao et al., 2022).

Habitat levels were classified using the Jenks natural breaks classification method, which looks for natural patterns in the data rather than artificial and rigid groupings (Chen et al., 2013). Jenks natural breaks classification classifies habitats into four categories: (1) inappropriate habitats (IH); (2) low-suitability habitats (LSH); (3) medium-suitability habitats (MSH); and (4) highly suitable habitats (HSH). To ensure that the future classification of habitat classes is consistent with the current distribution and allows for comparison, we applied the current grouping strategy of each model to the future distribution model so that the groupings were consistent and the grouping error was low to minimize the climate change uncertainty.

Using the current habitat as a criterion, habitat changes were classified into three broad categories based on the differences between the habitats in various climatic conditions and the current habitat: (1) areas with a constant habitat class over time; (2) areas with a decreased habitat class over time; and (3) areas with an increased habitat class over time.

Based on the current habitat classes, a statistical approach was used to determine trends in the rate of change in the area for various habitat classes under various climatic scenarios. By utilizing trends rather than simply increasing or decreasing the sizes of areas to classify conservation areas, the natural patterns of habitat change can be better understood to benefit human activities.

## Results

### Current distribution and future change trends

**FIGURE 2:**
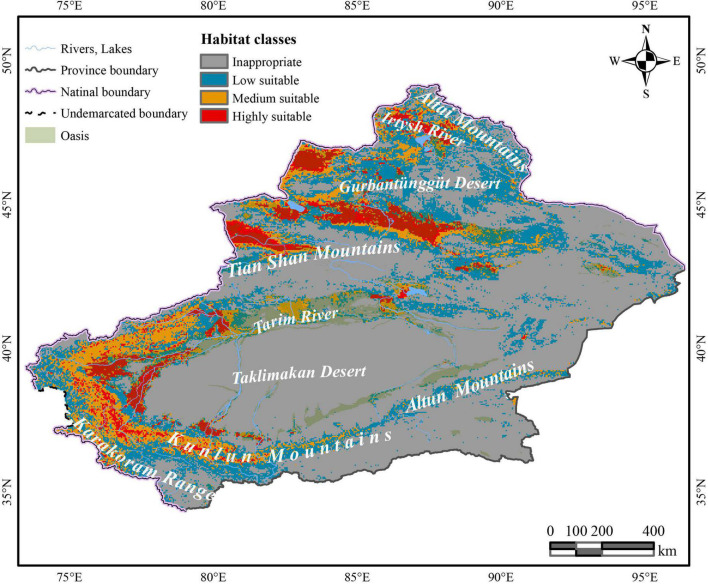
The predictive distribution of *C. salsa* with the main mountains and rivers in Xinjiang. The map is made based on the standard map no. GS(2017)1267 downloaded from the standard map service website of the Ministry of Natural Resources, without modification of the base map.

Under the effects of multiple host plants, it was predicted that *C. salsa* is distributed around the Xinjiang region (Figure 2). The HSH is mainly in the northern foothills of the Tian Shan Mountains, north of the Gurbantunggut Desert near the Irtysh River, and Tacheng Prefecture, which are all in the northern Xinjiang region. Additionally, in the southern Xinjiang region, HSH is predominantly located in the periphery of the Taklimakan Desert, and there are some HSH upstream of the Tarim River in the main oasis and in the northern foothills of the Karakoram Range. For the MSH, they are mainly located in the western Gurbantunggut Desert, upstream of the Tarim River, and in the northern foothills of the Karakoram Range. The LSH is in the eastern Gurbantunggut Desert and along the western margin of the Taklimakan Desert.

**FIGURE 3:**
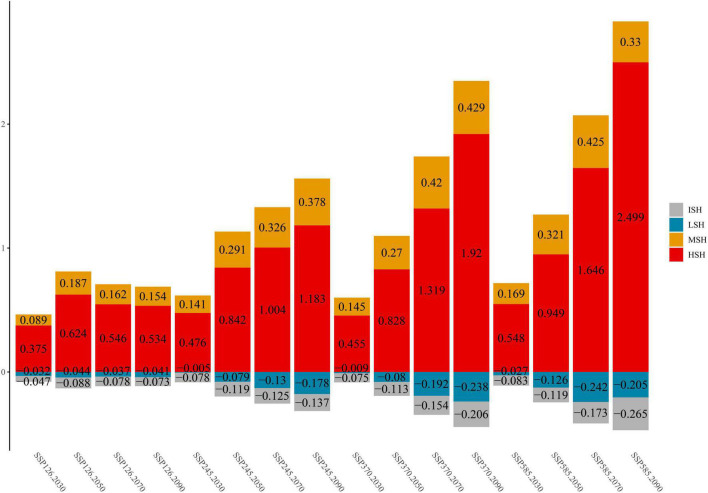
The change trends of *C. salsa* in each scenario (comparing the areas under current conditions, the positive value is increasing, and the negative value is decreasing). ISH, inappropriate suitable habitat; LSH, low-suitability habitat; MSH, medium-suitability habitat; HSH, highly suitable habitat. Additionally, 2,030, 2,050, 2,070, and 2,090 represent 2,020–2,040, 2,040–2,060, 2,060–2,080, and 2,080–2,100.

Additionally, the habitat change rate indicates the change in the *C. salsa* distribution under climate change conditions (Figure 3). In terms of the overall trend, MSH and HSH are always shown to increase in different climate scenarios. However, there are some differences between each scenario. In SSP126, the maximum growth ratio (MGR) of MSH appears from 2,040 to 2,060 (18.7%) and the MGR of HSH is 62.4% under the same scenario. After that period, the growth ratio gradually decreases. Additionally, for MSH, the same trend also appears in SSP585, with an MGR of 42.5% from 2,060 to 2,080. However, in other scenarios, the growth ratio of HSH still has an increasing trend, with an MGR of 249.9% in SSP585 from 2,080 to 2,100. In contrast to the increasing trend in the MSH and HSH, the LSH and ISH always show decreasing trends. Additionally, the maximum reduction ratio (MRR) of ISH is 26.5% in SSP585 for 2,080–2,100, and that of LSH is 24.2% in SSP585 for 2,060–2,080.

### The evaluations and uncertain of the models

**FIGURE 4:**
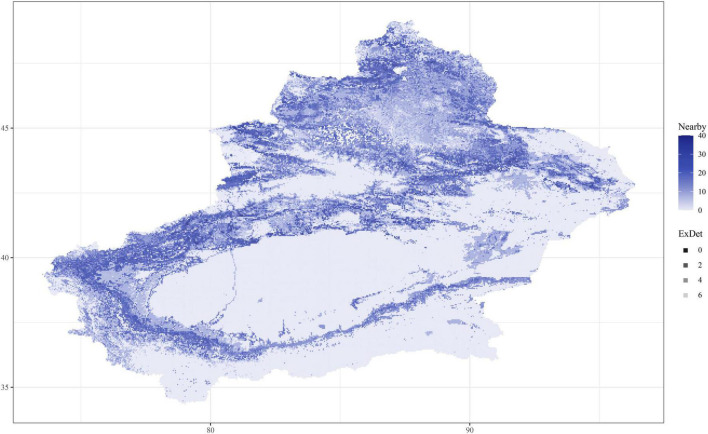
The uncertainty of *C. salsa* prediction. ExDet is the extrapolation detection metric, and Nearby is the percentage of data nearby (%N). When the color is deeper, the areas are more reliable (with high%N and low ExDet).

As shown in Table 1, the evaluations used in this study were AOC, CBI, and OR. In all of the models, the fundamental index, the CBI, is over 0.8; *K. ceratoides*, the maximum CBI is 0.88. Additionally, from the perspective of traditional evaluation methods, the AUC values of most of the models are over 0.8. However, the AUC of *K. cuspidatum* is 0.76. Additionally, the OR for all of the models is about 0.1, with the exception of S. physophora, where it is 0.15. For the uncertainity, according to alpha and color changes shown in Figure 4, a lower ExDet and a higher %N will result in a deeper color, and according to this, the most reliable areas can be easily observed. The least reliable areas are mainly located in maintained and desert zones, such as the Taklimakan Desert and Tianshan Mountains. The most reliable areas were located in the foothills of mountains, in the peripheries of the Taklimakan Desert and Gurbantunggut Desert. Compared to the current distribution and for the habitat classes, in particular, the most reliable areas overlap with HSH and MSH, such as the western edge of the Taklimakan Desert.

**TABLE 1:** The average and standard deviation of AUC, CBI, and the omission rate (OR).

	AUC avg	AUC SD	CBI avg	CBI SD	OR avg	OR SD
*A. splendens*	0.88	0.03	0.86	0.04	0.10	0.07
*C. salsa*	0.85	0.05	0.82	0.09	0.12	0.03
*K. ceratoides*	0.83	0.03	0.88	0.07	0.10	0.08
*K. cuspidatum*	0.76	0.03	0.82	0.08	0.09	0.09
*K. foliatum*	0.88	0.08	0.85	0.08	0.10	0.12
*K. gracile*	0.82	0.15	0.80	0.07	0.06	0.13
*N. sibirica*	0.84	0.04	0.81	0.07	0.10	0.08
*R. soongarica*	0.87	0.06	0.81	0.03	0.10	0.19
*S. physophora*	0.88	0.06	0.84	0.07	0.15	0.13

### The effects and changes of host plants in identifying reserves

**FIGURE 5:**
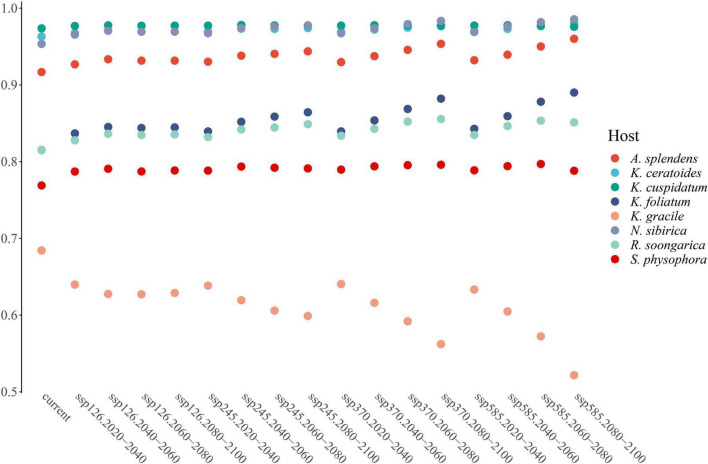
The niche similarity of host plants.

From the perspective of niche similarity (Figure 5), it can be seen that different host plants result in different trends in different climatic situations. The host plant *K. gracile*, for example (Figure 5), showed a higher value (0.684) in the current scenario than in any other climatic scenario, while the host plant *A. splendens* showed a steady increase in its value with time in other climatic scenarios. The distribution of host plants with mean values above 0.9 in different climatic conditions and time dimensions are considered the main influencing factors. The value of *K. cuspidatum* is about 0.977 and the value of *N. sibirica* is 0.973, near that of *K. cuspidatum*. Additionally, not only are these two host plants similar in terms of the evaluation of niche similarity, but four host plants, including the other two host plants, *K. ceratoides* (0.972), and *A. splendens* (0.938), can be regarded as effective factors. These plants not only have the highest niche similarity but also have the most influential covariates (MIC) for niche extrapolation. The results of the extrapolation are summarized in Table 2. For the extrapolation, the univariate plays a big role in *C. salsa* prediction, with a value of 73.77%. Additionally, for the MIC, *N. sibirica, K. ceratoides*, and *K. cuspidatum* occupy approximately 70% of all of the extrapolation results. Additionally, according to the habitat trends of the four host plants in different climatic situations, we can roughly divide them into two categories: (1) each major habitat (HSH, MSH) showed an increasing trend, including in the two host plants *A. splendens* and *K. cuspidatum* (Table 3); and (2) the major habitats showed a shrinking trend, including in *N. sibirica* and *K. ceratoides* (Table 3).

**TABLE 2:** The extrapolation values and the most influential covariates.

Table:	Extrapolation		
Type	Count	Percentage	
Univariate	73,980	73.77	
Combinatorial	6,696	6.68	
Sub-total	80,676	80.44	
Analogue	19,612	19.56	
Total	100,288	100	

**Table:**	**Most influential covariates (MIC)**		
**Type**	**Covariate**	**Count**	**Percentage**

Univariate	*N. sibirica*	33,594	33
Univariate	*K. ceratoides*	18,555	19
Univariate	*K. cuspidatum*	16,999	17
Univariate	*A. splendens*	2,118	2.1
Univariate	*K. gracile*	1,106	1.1
Univariate	*R. soongarica*	1,053	1
Univariate	*S. physophora*	460	0.46
Univariate	*K. foliatum*	95	0.095
Sub-total		73,980	74
Combinatorial	*S. physophora*	1,633	1.6
Combinatorial	*K. gracile*	1,192	1.2
Combinatorial	*R. soongarica*	1,111	1.1
Combinatorial	*K. foliatum*	872	0.87
Combinatorial	*N. sibirica*	697	0.69
Combinatorial	*A. splendens*	491	0.49
Combinatorial	*K. ceratoides*	441	0.44
Combinatorial	*K. cuspidatum*	259	0.26
Sub-total		6,696	6.7
Total		80,676	80

**TABLE 3:** The change trends of the host plants under different scenarios.

Scenarios	*A. splendens*				*K. cuspidatum*				*K. ceratoides*			*N. sibirica*				
	ISH	LSH	MSH	HSH	ISH	LSH	MSH	HSH	ISH	LSH	MSH	HSH	ISH	LSH	MSH	HSH
SSP126 2,020–2,040	−0.07	0.03	0.09	0.60	−0.13	−0.02	0.17	0.31	0.09	−0.09	−0.06	−0.17	−0.06	−0.03	−0.05	0.35
SSP126 2,040–2060	−0.12	0.07	0.18	0.95	−0.20	−0.03	0.24	0.46	0.04	−0.05	0.02	−0.10	−0.08	−0.03	−0.08	0.47
SSP126 2,060–2,080	−0.11	0.06	0.17	0.90	−0.20	−0.01	0.24	0.43	0.07	−0.07	−0.03	−0.18	−0.08	−0.03	−0.08	0.46
SSP126 2,080–2,100	−0.11	0.07	0.17	0.89	−0.18	−0.02	0.22	0.41	0.07	−0.06	−0.04	−0.18	−0.07	−0.03	−0.08	0.44
SSP245 2,020–2,040	−0.09	0.06	0.14	0.72	−0.17	−0.02	0.21	0.37	0.04	−0.04	0.00	−0.12	−0.07	−0.02	−0.06	0.40
SSP245 2,040–2,060	−0.16	0.09	0.27	1.26	−0.25	−0.03	0.28	0.61	−0.01	−0.01	0.07	−0.02	−0.10	−0.03	−0.12	0.61
SSP245 2,060–2,080	−0.18	0.08	0.30	1.49	−0.31	0.01	0.29	0.73	0.01	−0.03	0.04	−0.05	−0.11	−0.03	−0.15	0.72
SSP245 2,080–2,100	−0.21	0.06	0.37	1.80	−0.36	0.03	0.28	0.85	0.00	−0.02	0.04	−0.01	−0.12	−0.04	−0.17	0.80
SSP370 2,020–2,040	−0.09	0.07	0.14	0.71	−0.16	−0.03	0.21	0.36	0.05	−0.04	−0.01	−0.14	−0.07	−0.02	−0.06	0.40
SSP370 2,040–2,060	−0.16	0.09	0.25	1.25	−0.27	−0.02	0.29	0.62	0.03	−0.04	0.03	−0.09	−0.11	−0.03	−0.12	0.65
SSP370 2,060–2,080	−0.23	0.05	0.41	1.98	−0.42	0.07	0.28	0.99	−0.01	−0.01	0.06	−0.01	−0.14	−0.05	−0.19	0.91
SSP370 2,080–2,100	−0.30	−0.01	0.53	2.88	−0.62	0.24	0.23	1.45	−0.05	−0.01	0.07	0.19	−0.18	−0.06	−0.24	1.15
SSP585 2,020–2,040	−0.11	0.07	0.17	0.85	−0.18	−0.04	0.22	0.42	0.04	−0.05	0.00	−0.13	−0.07	−0.03	−0.08	0.45
SSP585 2,040–2,060	−0.18	0.08	0.29	1.49	−0.31	0.00	0.30	0.71	0.02	−0.03	0.01	−0.09	−0.11	−0.04	−0.15	0.71
SSP585 2,060–2,080	−0.26	0.00	0.48	2.44	−0.52	0.17	0.25	1.22	−0.02	−0.02	0.03	0.10	−0.16	−0.06	−0.22	1.04
SSP585 2,080–2,100	−0.39	0.10	0.45	3.82	−0.74	0.24	0.20	1.90	−0.07	−0.03	0.09	0.36	−0.21	−0.07	−0.26	1.33

**FIGURE 6:**
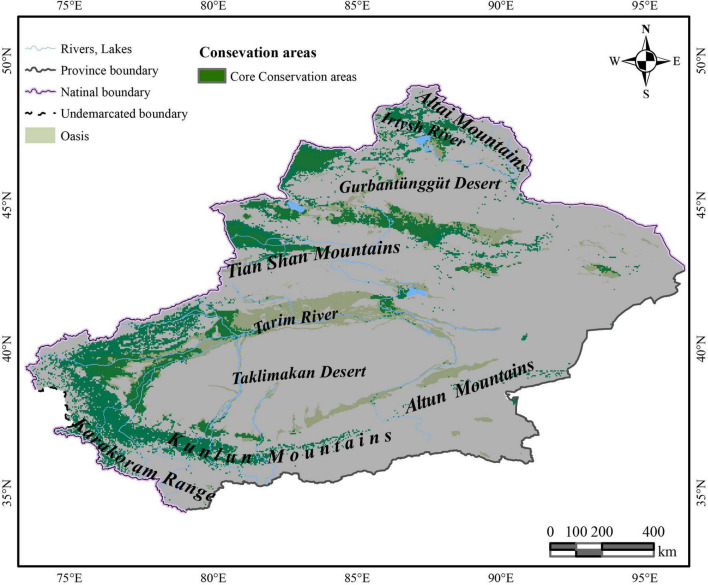
The identified nature conservation area of *C. salsa*.

In the increasing category, the MRR of *A. splendens* in HSH and MSH were found to be at 382.1% in SSP585 from 2,080 to 2,100 and 47.9% for SSP585 from 2,060 to 2,080. Additionally, the MRR of *K. cuspidatum* in HSH and MSH was 190.2% according to SSP585 from 2,080 to 2,100 and 29.6% in SSP585 from 2,040 to 2,060. For the host plants, in the decreasing category, the MSH of the host plant *N. sibirica* decreased, with a maximum reduction ratio of 26.1%. Additionally, the host plant *K. ceratoides* showed a very dangerous habitat change trend when the HSH of other host plants was increasing, and the HSH decreased in most cases, with a maximum decrease ratio of 17.7%. When identifying the natural reserves, the host plants with shrinking habitats should be focused, especially *N. sibirica* and *K. ceratoides*, in which habitat shrinkage was determined to be gradually increased. Therefore, according to the variations in the host plants in different climatic situations from the perspective of niche similarity and the influence of host plants from the perspective of MIC, and because of the variation in the parasitic plant *C. salsa*, the main strategy for identifying natural reserve is to focus on the main areas experiencing habitat shrinkage in both host and parasite distributions, including in the HSH of *K. ceratoides*, the MSH of *N. sibirica* and *K. ceratoides*, and the MSH and HSH of *C. salsa* under the current conditions. These areas are regarded as core protection zones (Figure 6), and the total area is about 126,483.5 km^2^.

## Discussion

When identifying natural reserves, the past studies have focused on a single change (the subject of research) in the climate (Li J. et al., 2019; Sun X. et al., 2020; Tang et al., 2021). This is a reliable method for non-parasite plants or other species; however, it is not suitable for parasitic and especially holoparasitic plants (Shao et al., 2022). For parasitic plants, biotic factors play a fundamental role in parasitic plants, especially in holoparasite plants (Dibong et al., 2012; Sayad et al., 2017; Hu et al., 2020). Particularly, Watson (2009) proposed the host-quality hypothesis, which is based on a summary of previous research and states that host quality is the critical factor governing the survival of parasitic plants. Follow-up studies on the host quality hypothesis have also focused on hemiparasitic plants, such as mistletoe and Loranthaceae (Dibong et al., 2012; Sayad et al., 2017). In studies of holoparasitic root plants, it has been shown that the host also has a relatively important influence on the growth of parasitic plants (Hu et al., 2020). Hence, from this point, the effects of host plants on *C. salsa* distribution prediction are fundamental factors in this study.

Additionally, for the multiple host plants, analyzing the effects of the hosts in the prediction process is the most important factor in reserve identification. Two approaches can be applied in reserve identification to evaluate the effects of hosts: niche overlap and MIC. Niche overlap simply indicates that there is competition between two or more species sharing the source in the same area (Pianka, 1974; Jakob et al., 2010; Sales et al., 2022). Additionally, if we bring the parasitic relationship between holoparasitic plants and host plants into the niche overlap theory, then the niche overlap value can be taken as the effect of host plant distribution on the distribution of parasitic plants (Jakob et al., 2010; Broennimann et al., 2012; Casadesús and Munné-Bosch, 2021). According to this, what niche overlap represents is not the competitive relationship between host and parasitic plants, but rather the similarity in their distribution (Li et al., 2014; Pili et al., 2020). In this view, the influence of the host plant on the predicted distribution of *C. salsa* was reflected by the niche overlap value, especially when we chose the host factor as the limiting factor.

Additionally, the effects of host distribution on extrapolation evaluation indicate more quantitative information, and the ExDet metric can show the MIC (for this study, there were different host distributions), which can make the largest contribution to extrapolation in the target system (Mesgaran et al., 2014; Bouchet et al., 2019). Additionally, univariate extrapolation is known as the mathematical, strict, novel, or type 1 extrapolation (implementing Mahalanobis distance), which can identify whether any given covariate is out of range and can be successfully applied for habitat suitable modeling (Clark et al., 1993; Farber and Kadmon, 2003; Mesgaran et al., 2014).

**FIGURE 7:**
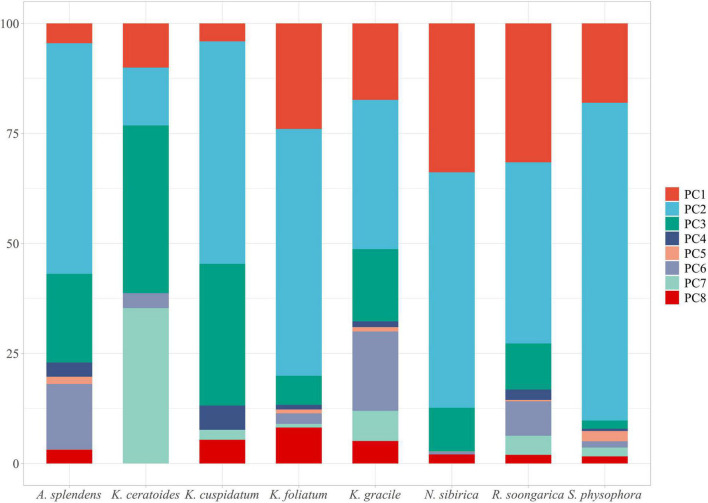
The main effect factors in host plants predictions.

Additionally, according to the MIC table (Table 2), the host plants *N. sibirica, K. ceratoides*, and *K. cuspidatum* play a role in parasite distribution prediction, especially *N. sibirica*. The ExDet metric can be regarded as a tool for considering prior covariates, and in this study, the effects of *N. sibirica* and the other two host plants are fundamental in production model processing or data extrapolation (Guisan et al., 2017; Mahony et al., 2017; Yates et al., 2018; García-Barón et al., 2019). Additionally, based on the effects of the hosts, climate change is a popular factor in MaxEnt modeling for plants. In this study, the climate changes play an indirect role in the predicted process. Figure 7 is showing the different factors with their effect-percentage in host plants predicted process. For example, the host plant *N. sibirica* is mainly affected by PC2 and PC1, and these two components contain more than two environmental variables’ features, every environmental variable can be affected by climate changes. And especially in an arid land, water, especially groundwater, plays an important role in plant growth (Xiong et al., 2018; Yang et al., 2020). As such, changes in the climate, including temperature and water changes, are regarded as the basis of natural reserve identification (Yue et al., 2021). Therefore, in this study, the final protected zone is with a combination of the effects of all the selected hosts from the predictions, the main hosts’ effects for reserve identification, and the effects of future climate change trends.

However, our study cannot effectively explain the factors causing different desert plants to have different climate change trends and cannot effectively explore how the deeper groundwater affects the arid plants’ distribution. In our future study, better explanatory variables will be discovered and introduced in addition to the hosts and environment in order for the results of the evaluation to be more accurate.

## Conclusion

A few host plants play fundamental roles in distribution prediction. The host plants *A. splendens, K. cuspidatum, N. sibirica*, and *K. ceratoides* are more important than other host plants from the perspective of niche similarity. Additionally, considering the MIC, *K. ceratoides* and *N. sibirica* are fundamental factors in core natural reserve identification. To identify natural reserves, especially under climate change conditions, multiple host parasitic plants require more attention to determine the effects of their host plants. For *C. salsa*, its host plants *N. sibirica* and *K. ceratoides* can provide good aid for planning and protecting areas. Under the same conditions of good similarity and MIC, the trends of the host habitat can likewise affect the future distribution of *C. salsa*, similar to the role of environmental variables in the prediction of non-parasitic plants. The MSH of *N. sibirica* and *K. ceratoides* and the HSH of *K. ceratoides* are combined with the MSH and HSH of *C. salsa*, which can better demonstrate the accuracy of identifying areas from the perspective of the host–parasite relationship. The core area for *C. salsa* was determined to have a final area of 126483.5 km^2^.

## Data availability statement

The original contributions presented in this study are included in the article/supplementary material, further inquiries can be directed to the corresponding author.

## Author contributions

MS: investigation, data curation, methodology, formal analysis, and writing—original draft. JF: conceptualization, methodology, software, writing—review and editing, funding acquisition, investigation, project administration, resources, and supervision. JM and LW: validation and writing—review and editing the manuscript. All authors contributed to the article and approved the submitted version.
